# Blood-brain barrier water exchange in relation to amyloid, cognition and cerebrovascular burden

**DOI:** 10.1016/j.nicl.2025.103926

**Published:** 2025-12-11

**Authors:** Beatriz E. Padrela, Sandra Tecelão, Bjørn-Eivind Kirsebom, Oliver Geier, Mario Tranfa, Federico Masserini, Markus H. Sneve, Maksim Slivka, Emilie Sogn Falch, Lene Pålhaugen, Amnah Mahroo, Klaus Eickel, David L. Thomas, Matthias Günther, Per Selnes, Atle Bjørnerud, Kristine B. Walhovd, Anders M. Fjell, Frederik Barkhof, Jan Petr, Tormod Fladby, Henk J.M.M. Mutsaerts

**Affiliations:** a Department of Radiology and Nuclear Medicine, Amsterdam University Medical Center, Location VUmc, Amsterdam, the Netherlands; b Brain Imaging, Amsterdam Neuroscience, Amsterdam, the Netherlands; c Department of Neurology, Akershus University Hospital, Oslo, Norway; d Department of Neurology, University Hospital of North Norway, Tromsø, Norway; e Department of Psychology, Faculty of Health Sciences, UiT The Arctic University of Norway, Tromsø, Norway; f Computational Radiology and Artificial Intelligence, Division of Radiology and Nuclear Medicine, Oslo University Hospital, Oslo, Norway; g Center for Lifespan Changes in Brain and Cognition, Department of Psychology, University of Oslo, Oslo, Norway; h Institute of Clinical Medicine, Campus Ahus, University of Oslo, Oslo, Norway; i Fraunhofer-Institute for Digital Medicine MEVIS, Bremen, Germany; j Bremerhaven University of Applied Sciences, Bremerhaven, Germany; k Department of Brain Repair and Rehabilitation, UCL Queen Square Institute of Neurology, University College London, London, UK; l Centre for Medical Image Computing (CMIC), University College London, London, UK; m MR-Imaging and Spectroscopy, University of Bremen, Bremen, Germany; n mediri GmbH, Heidelberg, Germany; o Helmholtz-Zentrum Dresden-Rossendorf, Institute of Radiopharmaceutical Cancer Research, Dresden, Germany

**Keywords:** Blood-brain barrier water exchange, Arterial spin labeling, Cerebral blood flow, Amyloid, Cognition, Cerebrovascular damage

## Abstract

Blood-brain barrier (BBB) water exchange may serve as a sensitive early biomarker for Alzheimer’s disease and age-related cognitive decline. This study applied a non-invasive multi-echo arterial spin labeling (ASL) technique to measure BBB water exchange time (Tex), cerebral blood flow (CBF), and arterial transit time (ATT) in 160 adults aged 50 years and older. Participants were classified as cognitively normal (CN), having subjective cognitive decline (SCD), or mild cognitive impairment (MCI). They were assessed for amyloid status and cerebrovascular burden. Compared to CN participants, Tex was significantly lower in both SCD (−9.5 %) and MCI (−14.5 %) groups, suggesting that reductions in BBB water exchange emerge early in the course of cognitive decline. In contrast, CBF was reduced only in MCI participants (−20.8 % compared to CN), and ATT was significantly increased only in individuals with severe cerebrovascular burden (Fazekas score 3). Notably, Tex showed a stepwise decrease with increasing Fazekas scores (1–2), supporting its sensitivity to moderate small vessel disease. No associations were found between Tex and amyloid positivity after adjusting for age and sex. These findings indicate that Tex alterations may precede changes in traditional perfusion markers and are more closely related to vascular and early cognitive changes than to amyloid pathology. BBB water exchange mapping may therefore provide a promising, non-invasive tool to detect early neurovascular dysfunction that contributes to cognitive decline in aging populations, potentially offering a useful biomarker for early intervention trials targeting vascular contributions to dementia.

## Highlights

• BBB water exchange time (Tex) is reduced in early cognitive impairment.
• Tex decreases with moderate white matter hyperintensities burden.
• Tex changes precede hemodynamic changes in cognitive and cerebrovascular decline.
• These Tex changes are independent of amyloid status after age and sex adjustment.

## 1 Introduction

Blood-brain barrier (BBB) dysfunction is increasingly recognized as an early neurovascular event in Alzheimer’s Disease (AD) (Alkhalifa, 2023, Kurz et al., 2022), contributing to neurodegeneration (Uchida et al., 2023). Studies using gadolinium-based contrast agents (GBCA) have demonstrated increased BBB permeability in individuals with mild cognitive impairment (MCI) compared to healthy controls (Li et al., 2021), suggesting that early disruptions in BBB integrity may precede clinical symptoms of cognitive decline. However, the cost and concerns about safety(Kanda et al., 2016) of GBCAs limit its feasibility for screening and underscore the need for non-invasive techniques to investigate BBB exchange dynamics.

Endogenous water labeled using arterial spin labeling (ASL) can serve as a sensitive, non-invasive tracer to detect subtle and early changes in BBB permeability (Mahroo et al., 2023). Changes in BBB water exchange have been linked to aging and AD in both animal (Dickie et al., 2021, Ohene et al., 2020, Dickie, 2019, Ohene, 2025, Xiong, 2025) and human (Mahroo et al., 2023, Ford, 2022, Pappas, 2024, Lin, 2021, Gold, 2021) studies, suggesting its potential role in amyloid clearance and early pathophysiology of AD. To date, however, little attention has been devoted to the role of BBB water dynamics in the early stages of AD, particularly in relation to well-established AD biomarkers (Lin, 2021).

An emerging technique that obviates the need for exogenous contrast agents is multiple echo time arterial spin labeling (multi-TE ASL) (Mahroo, 2021, Gregori et al., 2013), which can noninvasively quantify BBB water exchange. Unlike traditional single-compartment ASL models, multi-TE ASL distinguishes between water molecules in intravascular and extravascular compartments based on differences in T2 relaxation times (Clement, 2022). This difference allows for the estimation of the water exchange time (Tex) between the compartments to quantify BBB water exchange. Pilot studies have demonstrated the sensitivity (Ohene, 2019), reproducibility (Mahroo, 2021); and feasibility of this multi-TE ASL sequence to study BBB water exchange (Mahroo et al., 2023); and aging results are available in mice (Ohene et al., 2020) and humans (Mahroo et al., 2023, Shao, 2019, Padrela, 2025). However, the use of multi-TE ASL to investigate Tex changes in age-related neurodegeneration has not yet been explored.

The “DEveloping BBB-ASL as non-Invasive Early biomarker” (DEBBIE) (Padrela, 2024) project has refined this technique to quantify Tex alongside hemodynamic parameters such as cerebral blood flow (CBF) and arterial transit time (ATT). These metrics provide insights into the underlying mechanisms that may lead to impaired blood delivery, which is common in patients with cerebrovascular comorbidities (Mutsaerts, 2020). Most importantly, this technique has been optimized to acquire these parameters within a clinically feasible scanning time, and has shown to be reproducible (Mahroo, 2021, Mayaert, et al.,) **[R.1.1.]** allowing large studies to investigate the role of BBB water exchange as a disease biomarker and potentially for monitoring disease-modifying treatment (Mahroo, 2021).

The present work extends the use of BBB-ASL to the AD continuum in a cohort of 160 participants. To probe the potential link between AD pathophysiology and BBB water permeability (Li et al., 2021, Zhang, 2023), this study investigates whether Tex is associated with 1) amyloid status (measured by PET or lumbar puncture), 2) cognitive staging, or 3) white matter hyperintensities (WMH) burden as a radiological marker commonly associated to cerebrovascular damage, both globally and regionally in vascular territories and AD-specific areas. To track these changes along the AD continuum, we studied cognitively normal (CN) participants and participants with subjective cognitive decline (SCD) and mild cognitive impairment (MCI) (Wang, 2020). We additionally explored CBF and ATT as reference hemodynamic parameters.

## 2 Materials and Methods

### 2.1 Study participants

Data were drawn from the Center for Lifespan Changes in Brain and Cognition (LCBC) and the Dementia Disease Initiation (DDI) (Fladby, 2017) cohorts, all scanned with the same MRI scanner and identical ASL MRI protocol in Oslo, Norway, between October 2022 and November 2023. The LCBC sample is a population-based cohort including CN participants, while DDI is an outpatient memory clinic cohort including CN, SCD, and MCI (Fladby, 2017) participants. The DDI CN participants were recruited from advertisements and memory clinic referrals with a family history of dementia, or as cohabitants of patients with dementia.

SCD classification was based on participants’ self-reported cognitive decline despite normal performance on neuropsychological tests. Participants answered structured, binary-response questions assessing perceived cognitive decline, related concerns, and functional impact. These responses, collected via the DDI case report form, aligned with standard SCD diagnostic frameworks (Jessen, 2014); and self-report reliability was assumed given normal cognitive test results.

MCI diagnosis followed the criteria by Fladby et al. (2017) (Fladby, 2017) based on a T-score ≤ 35 on at least one of the following: the CERAD word list recall, Trail Making Test-B (TMT-B), Controlled Oral Word Association (COWAT), or the Visual Object and Space Perception (VOSP) silhouettes subtests (Albert, 2011). Exclusion criteria for both cohorts included a history of brain trauma or disorder, clinical stroke, diagnosed dementia, severe psychiatric illness, or other neurodegenerative conditions likely to affect cognition (Fladby, 2017, Wang, 2022).

### 2.2 MRI acquisition and processing

All participants were scanned on the same 3 T MRI scanner (MAGNETOM Prisma, Siemens Healthineers, Erlangen, Germany) using a 32-channel head coil (Mahroo, 2021, Gregori et al., 2013).

For LCBC, 3D T1-weighted Magnetization Prepared RApid Gradient Echo (MPRAGE) image was acquired with the following parameters: TR = 2400 ms, TE = 2.22 ms, inversion time (TI) = 1000 ms, flip angle = 8°, FOV = 256 x 240 mm^2^, spatial resolution = 0.8 x 0.8 x 0.8 mm^3^, matrix size = 320 x 300, number of slices = 208, AC-PC aligned sagittal orientation. A 2D coronal FLAIR (0.43x 0.43x4 mm^3^ voxels) was acquired with TR = 9000 ms, TE = 87 ms, TI = 2500 ms, FOV = 220x220 mm^2^, 39 slices, slice gap = 1.2 mm.

The DDI cohort protocol included a 3D MPRAGE (1 mm^3^ isotropic voxels), with TR = 2200 ms, TE = 1.47 ms, TI = 900 ms, FOV = 256x256 mm^2^, 224 slices. A 3D SPACE FLAIR (1 mm^3^ isotropic voxels), TR = 5000 ms, TE = 388 ms, TI = 1800 ms, T2 variable FA mode, FOV = 256x256 mm^2^, 224 slices. Both scans were acquired with an AC-PC aligned sagittal orientation.

BBB-ASL data were acquired using the Fraunhofer MEVIS-developed DEBBIE sequence, combining two Hadamard-encoded pseudo-continuous arterial spin labeling (pCASL) protocols [16 ]with a 3D Gradient and Spin Echo (GRASE) readout (Günther et al., 2005). Full technical details of the sequence design and optimization can be found in a previous publication (Mahroo, 2021). This two-sequence setup enables improved estimation of cerebral blood flow (CBF) and arterial transit time (ATT) (single-echo Hadamard-8 (HAD8) sequence), and blood–brain barrier water exchange time (Tex) (multi-echo Hadamard-4 (HAD4) sequence). The HAD8 sequence included two separate measurements with different PLD sets: PLDs = [600, 1000, 1400, 1800, 2200, 2600, 3000] ms and PLDs = [800, 1200, 1600, 2000, 2400, 2800, 3200] ms, each with a sub-bolus duration (SBD) of 400 ms, TE = 13.4 ms, TR = 4190 ms, one segment, and a scan duration of 2:18 min. The HAD4 sequence for Tex estimation used PLDs = [500, 1500, 2500] ms, SBD = 1000 ms, eight echo times (TE = 14:28:210 ms), TR = 4670 ms, two repetitions, six segments, and a scan duration of 3:49 min. Both sequences used frequency-offset corrected inversion (FOCI) pulses for background suppression, timed to suppress signal contributions with T1 values of 700 ms and 1400 ms. A separate M0 image (TR = 5000 ms, no labeling or background suppression) was acquired for quantification. The total scan time for ASL and M0 imaging was approximately 7 min.

Datasets were analyzed with ExploreASL(Mutsaerts, 2020) version 1.12 beta, commit 0d72417, using default settings. This included segmentation and spatial normalization of the T1-weighted images to MNI space with Computational Anatomy Toolbox 12(Gaser, 2009); as well as affine registration of ASL to T1-weighted images.

#### 2.2.1 Cerebrovascular burden

WMH load was quantified using the Fazekas score (Fazekas et al., 1987). For DDI, Fazekas scores were assessed by a neuroradiologist as part of the usual clinical workup. For LCBC, Fazekas scores were assessed by two neuroradiologists (MT and FM, with seven and nine years of experience, respectively).

#### 2.2.2 ASL quantification

The Hadamard-encoded ASL signal at each PLD and TE was decoded. The decoded images from both sequences were concatenated, and data were fitted voxelwise in a single step using FSL FABBER (version 6.0.4), adapted for Tex quantification as described previously (Mahroo, 2021, Chappell et al., 2009). Briefly, an extended multi-TE exchange model was used to estimate Tex, representing the mean residence time of water in the capillary compartment as a proxy for BBB water exchange. In each voxel, the signal was assumed to arise from three components: (1) arterial blood, (2) intravascular water at the capillary exchange site, and (3) extravascular tissue water following BBB exchange. The model used literature-based relaxation values: T2_blood_ = 165 ms, T2_tissue_ = 85 ms, T1_blood_ = 1650 ms, and T1_tissue_ = 1300 ms. Signal decay across eight echo times was used to capture the differential relaxation of each compartment. For details of the underlying biophysical model, see (Mahroo, 2021). An example of the modeled signal decay and its decomposition into vascular and tissue components is shown in Supplementary Fig. 1.

#### 2.2.3 Quality control (QC)

**Fig. 1:**
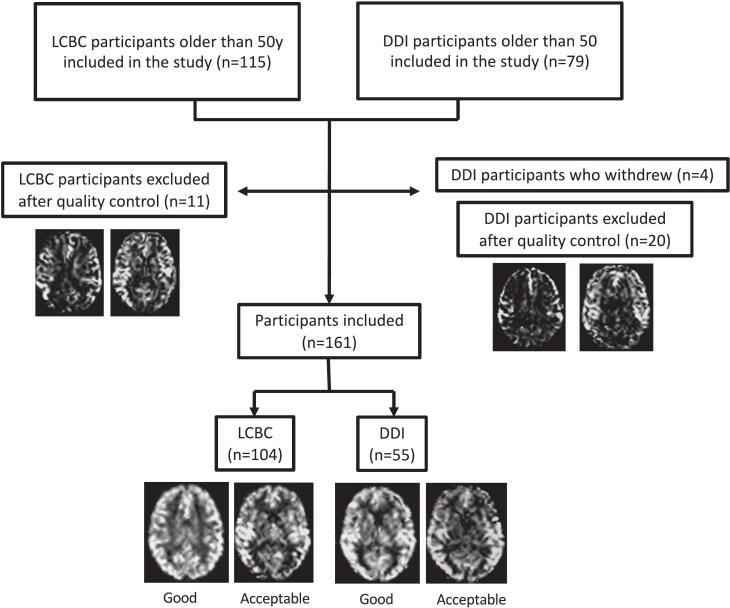
Inclusion and exclusion of participants for analysis based on quality control of CBF maps. Example CBF maps are shown. CBF: cerebral blood flow.

Since Tex maps show little contrast, we performed visual quality control using the associated CBF maps. Two authors (B.P. and H.M.) — with 4 and 10 years of ASL experience, respectively — independently performed visual inspection of the CBF maps. Participants with major motion artifacts, incomplete label arrival, or poor tissue contrast were excluded. Discrepancies were resolved by consensus. Analyses were conducted using images from participants with acceptable or good image quality (Fig. 1) (Mutsaerts, 2019).

#### 2.2.4 Region-of-interests (ROIs)

We defined ROIs by intersecting the total GM mask with anatomical masks in MNI space: (1) Total GM, (2) vascular territory ROIs — including anterior and posterior circulation regions and their ratio, with the anterior circulation ROI calculated using weighted average of the ACA and MCA ROIs from Tatu atlas (Tatu et al., 1998) taking into account the ROI size in voxels; and (3) three AD-specific ROIs associated with BBB dysfunction: precuneus, frontal cortex, and anterior and posterior cingulate cortex (Meng, 2023, Miners et al., 2018, Lee, 2020). We avoided using smaller ROIs such as the hippocampus, which may require further validation with this relatively new acquisition.

### 2.3 Assessment of amyloid status

Amyloid status was assessed using either PET or cerebrospinal fluid (CSF) sampling via lumbar puncture; the categorization of each participant as amyloid positive (A + ) or negative (A-) was performed according to the local procedures for each cohort.

For LCBC, all subjects underwent an [^18^F]Flutemetamol amyloid-PET scan to assess cortical Aβ burden (Wang, 2022). Images were acquired on a GE HealthCare Discovery PET/CT 690 scanner at Aleris Hospital and Radiology, Oslo, Norway. PET images were processed using PetSurfer, the MRI-PET analysis tool within the FreeSurfer (v7.1.0) package. The intensities were processed with Region-Based Voxel-wise Partial Volume Correction (RBV-PVC) and scaled by the cerebellar cortex (left and right). The resulting uptake values were then further analyzed with the sklearn (v1.5.1) toolbox. Principal component analysis was used to extract the first principal component (PC1) from the standardized uptake value ratios (SUVR) in the region of interest described by Mormino et al. (2014) (Mormino, 2014). Next, assuming data normality, PC1 was used to estimate the parameters of the Gaussian Mixture Model (GMM) with two mixture components; thus, two distributions were estimated. This allowed the probability of each observation belonging to each distribution to be calculated. Those falling under the distribution with the higher mean were classified as A + participants.

For DDI, CSF obtained through a lumbar puncture was used (n = 38), unless a participant refused lumbar puncture, in which case amyloid-PET was performed (n = 12). Amyloid status was defined as A + or A- from the CSF amyloid-beta 42/40 ratio (cut-off ≤ 0.077) (Siafarikas, 2021) or amyloid-PET by visual read, when available. [^18^F]Flutemetamol amyloid-PET scans were used to image cortical Aβ burden. PET scans were performed using a Philips Ingenuity Time-of-Flight PET–MRI scanner. Images were also visually rated according to the GE HealthCare reader guidelines (Buckley, 2017). Aβ status was rated by a trained neuroradiologist.

### 2.4 Statistical analysis

Associations of Tex, CBF, and ATT with age and sex, and the age*sex interaction, were tested for all participants to investigate covariate candidates. Independent two-sample t-tests were used to examine differences in Tex, CBF, and ATT based on amyloid status. For cognitive staging and Fazekas scores, one-way ANOVA was performed to assess group differences, followed by post-hoc Tukey Honestly Significant Difference tests.

Next, linear models were applied in a stepwise manner: first without covariates, then adjusting for age and sex, and finally including all covariates together (age, sex, amyloid positivity, cognitive staging, Fazekas scores). These models assessed ASL metrics differences between (1) A + and A- groups, (2) CN, SCD, and MCI, and (3) Fazekas score groups. In the amyloid status cases, the amyloid-negative group was used as the reference; CN was used as the reference for cognition, and Fazekas = 0 served as the reference for cerebrovascular burden analysis. These linear models were repeated for vascular territories and AD-specific regions. Only for the regional analysis were p-values corrected for multiple comparisons using the False Discovery Rate (FDR) method.

All statistical analyses were performed in R (v4.3.2; R Core Team 2023), with p < 0.05 considered statistically significant.

## 3 Results

### 3.1 Demographic characteristics

Out of the initial 194 participants, 160 were included after QC; 106 were from LCBC, and 54 were from DDI. Twelve participants were excluded based on visible head motion (n = 3 from LCBC and n = 9 from DDI) and 18 based on poor tissue contrast (sCoV > 0.8; n = 6 from LCBC, n = 12 from DDI) (Fig. 1). The final sample (Table 1) comprised 19 MCI participants and 18 A + participants, of whom 12 were both MCI and A + . The LCBC cohort included only cognitively normal (CN) participants, including 12 A + individuals. Table 1 presents the demographic characteristics of both cohorts, showing no significant age differences and a similar distribution of males and females. MMSE scores were also not significantly different; however, the quartile distributions differed, indicating that the DDI cohort had a broader range and lower scores in the lower quartile.

**Table 1:** Study sample demographics.

	**LCBC(n = 106)**	**DDI(n = 54)**	**Cohort difference (p-value)**	**All participants(n = 160)**
**Age (y)**				
Mean ± SD	64.9 ± 8.79	65.9 ± 7.37	0.428	65.0 ± 8.3
**Sex**				
Females (%)	70 (66 %)	33 (61 %)	0.659	103 (64 %)
**MMSE**				
Median (IQ range)	29 (29–30)	29 (27–30)	0.758	29 (28–30)
**Fazekas scores**				
0/1/2/3	51/37/14/4	9/34/9/2	0.0011**	60/71/22/6
**Cognition(Amyloid status)**				
CN (A-/A+/NA)	106 (94/12/0)	8 (7/0/1)	−	114 (101/12/1)
SCD (A-/A+/NA)	0	27 (19/6/2)	−	27 (19/6/2)
MCI (A-/A+/NA)	0	19 (6/12/1)	−	19 (6/12/1)

A: Amyloid positivity; CN: Cognitively normal; IQ: Interquartile range; MCI: Mild cognitive impairment; NA: Not available; SD: Standard deviation; WMH: White matter hyperintensities. Age comparisons were made using a *t*-test. Comparisons between cohorts for the number of males/females and the proportion of subjects in each Fazekas category were performed using the Chi-squared test. MMSE score comparisons were made using the Mann-Whitney *U* test (rank-based). For four participants in the DDI cohort, amyloid status could not be determined, as they declined both a spinal puncture and an amyloid PET scan. Additionally, if they had a prior amyloid status and it was negative and could not be extrapolated to the current visit.

### 3.2 Associations with age and sex

**Fig. 2:**
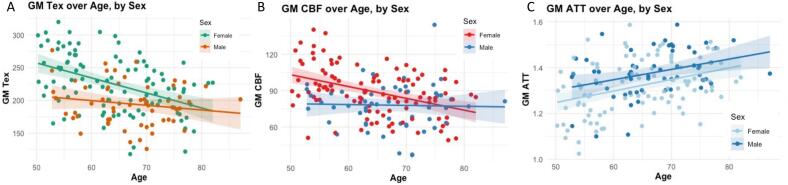
CBF (mL/100g/min), Tex (ms), and ATT (s) over age (A–C) with the respective model parameters for age, sex, and their interaction. *p < 0.05; **p < 0.01; ***p < 0.001. ATT: arterial transit time; CBF: cerebral blood flow; GM: gray matter; Tex: time of exchange. Sex is referenced to females, so a negative beta value means that males have a lower value compared to females.

The influence of age and sex on Tex, CBF, and ATT is shown in Fig. 2, with linear model results shown in Table 2. Both Tex and CBF were shown to be significantly influenced by age, sex, and their interaction (Fig. 2). Males show significantly lower Tex and CBF values than females, with a positive interaction term suggesting a weaker age-related decline in males. ATT (Fig. 2C) was significantly associated with age, showing an increase over time.

**Table 2:** CBF (mL/100 g/min), Tex (ms), and ATT (s) respective model parameters for age, sex, and their interaction. *p < 0.05; **p < 0.01; ***p < 0.001. ATT: arterial transit time; CBF: cerebral blood flow; GM: gray matter; Tex: time of exchange. Sex is referenced to females, so a negative beta value means that males have a lower value compared to females.

**Total GM Tex (ms)**	∼ Age + Sex + Age*Sex	
	*β*	*p*
Age	−2.29	<0.001***
Sex	−129.0	0.013*
Age * Sex	1.57	0.042*
**Total GM CBF (mL/100 g/min)**	∼ Age + Sex + Age*Sex	
	*β*	*p*
Age	−0.97	<0.001***
Sex	−69.1	0.008**
Age * Sex	0.90	0.019*
**Total GM ATT (s)**	∼ Age + Sex + Age*Sex	
	*β*	*p*
Age	5.02	<0.001***
Sex	80.9	0.572
Age * Sex	−0.52	0.806

### 3.3 Differences between amyloid, cognition and cerebrovascular burden stages

**Fig. 3:**
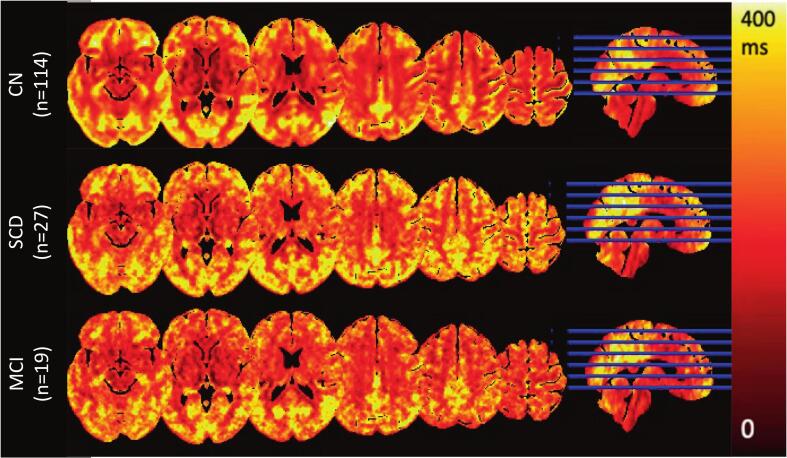
Group-average whole-brain Tex maps for the CN group (n=114, top row), SCD group (n=27, middle row) and MCI group (MCI, n=19, bottom row). CN: cognitively normal; MCI: mild cognitive impaired; SCD: subjective cognitive decline.

**Fig. 4:**
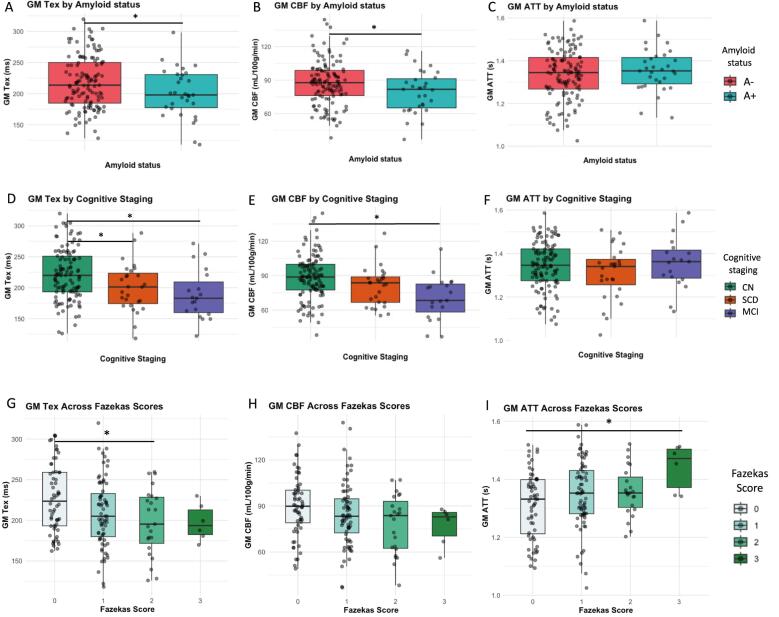
Investigating CBF, Tex, and ATT in amyloid status (A-C), cognitive staging (D-F), and vascular burden (G-I). Plots show differences without correction for age and sex. * p<0.05 for T-tests (A-C) and ANOVA (D-I) tests, respectively. ATT arterial transit time; CBF: cerebral blood flow; CN: cognitively normal; MCI: mild cognitive impairment; Tex: time of exchange.

Fig. 3 shows the average Tex maps of the whole-brain group for CN, SCD, and MCI participants. The same can be found in Supplementary Fig. 2 and 3 for CBF and ATT, respectively. Boxplots show the differences of GM Tex, CBF, and ATT between A + vs A− (Fig. 4 A-C), cognitive staging groups (Fig. 4 D-F), and Fazekas scores (Fig. 4 G-I). Differences were found between A + and A − for total GM Tex (p = 0.033) and GM CBF (p = 0.046), with lower values of both parameters in the A + group. No differences in GM ATT were observed related to amyloid status (p = 0.323). Group-wise GM Tex, CBF, and ATT values of each one of the groups can be found in Supplementary Table 1 for reference (see Fig. 4).

Regarding cognitive staging (Fig. 4 D-F), ANOVA revealed significant group differences for GM Tex (F = 7.027, p = 0.0012) and GM CBF (F = 8.306, p = 0.0037) but no significant difference for GM ATT (F = 1.128, p = 0.326). Post-hoc analyses showed significant differences in GM Tex between CN and SCD (β = –29.4 ms, p = 0.042) and CN and MCI (β = –32.6 ms, p = 0.0045), while GM CBF differed significantly only between CN and MCI (β = –18.5 mL/100 g/min, p = 0.0004). Between the Fazekas scores (Fig. 4 G-I), there were differences for GM Tex (F = 3.915, p = 0.009) and GM ATT (F = 3.413, p = 0.019) but not for GM CBF (F = 2.337, p = 0.08). Post-hoc analyses showed a borderline difference in GM Tex between Fazekas 0 and 1 (β = –18.55 ms, p = 0.055) and a significant difference between Fazekas 0 and 2 (Fig. 4G, β = –29.3, p = 0.023), and ATT differed significantly between Fazekas 0 and 3 (Fig. 4I, β = 0.13 s, p = 0.034).

Linear models revealed significantly lower Tex in A + compared to A- (β = –17.5 ms, p = 0.037), but this difference became non-significant after adjusting for age and sex (Supplementary Table 2). CBF showed a borderline association with amyloid status (β = –7.83 ms, p = 0.049), which also lost significance after age and sex adjustments (Supplementary Table 2).

Table 3 presents the linear models for Tex, CBF, and ATT associated with amyloid status, cognitive staging, and Fazekas scores, all adjusted for age and sex. Consistent with the boxplots (Fig. 4), both Tex and CBF remained significantly associated with cognitive staging, also after adjustments for age and sex (Table 3, Supplementary Table 2). Notably, Tex was significantly lower in SCD compared to CN, while CBF only differed between CN and MCI, and ATT showed no differences. ATT was significantly higher in Fazekas 3 compared to Fazekas 0, a finding that persisted after age and sex correction (Table 3, Supplementary Table 2).

**Table 3:** Linear models assessing the associations between global GM Tex (top rows), global GM CBF (middle rows) and global ATT (bottom rows) across different models are shown in different columns, all corrected for age and sex. P-values for amyloid status are reported relative to A-, while p-values for cognitive staging are relative to CN.

**Total GM Tex (ms)**	**∼ Amyloid status + age + sex**		**∼ Cognitive staging + age + sex**		**∼ Fazekas scores + age + sex**		**∼ All**	
	*β*	*p*	*β*	*p*	*β*	*p*	*β*	*p*
Amyloid status	−10.9	0.164					3.29	0.708
Cognitive staging (SCD)			−16.3	0.036*			−17.4	0.035*
Cognitive staging (MCI)			−31.7	<0.001***			−31.8	0.003**
Fazekas = 1					−13.4	0.045*	−6.72	0.325
Fazekas = 2					–23.5	0.013*	−16.9	0.073
Fazekas = 3					−13.6	0.405	−7.42	0.645

**Total GM CBF (mL/100 g/min)**	**∼ Amyloid status + age + sex**		**∼ Cognitive staging + age + sex**		**∼ Fazekas scores + age + sex**		**∼ All**	
	*β*	*p*	*β*	*p*	*β*	*p*	*β*	*p*

Amyloid status	−5.36	0.172					1.32	0.763
Cognitive staging (SCD)			−5.87	0.126			−5.60	0.172
Cognitive staging (MCI)			−18.14	<0.001***			−18.8	<0.001***
Fazekas = 1					−3.02	0.366	1.30	0.702
Fazekas = 2					−9.32	0.050	−7.48	0.114
Fazekas = 3					−6.16	0.45	−2.99	0.710

**Total GM ATT (s)**	**∼ Amyloid status + age + sex**		**∼ Cognitive staging + age + sex**		**∼ Fazekas scores + age + sex**		**∼ All**	
	*β*	*p*	*β*	*p*	*β*	*p*	*β*	*p*

Amyloid status	−0.005	0.983					<0.001	0.998
Cognitive staging (SCD)			−0.04	0.043*			−0.003	0.055
Cognitive staging (MCI)			−0.009	0.714			−0.004	0.996
Fazekas = 1					0.025	0.184	0.024	0.109
Fazekas = 2					0.028	0.290	0.059	0.184
Fazekas = 3					0.137	0.023*	0.156	0.007**

When including all variables in the same model (Table 3, rightmost column) — age, sex, amyloid status, cognitive staging, and Fazekas scores—Tex remained significantly different between SCD and CN (β = –17.4 ms, p = 0.035) and between MCI and CN (β = –31.8 ms, p = 0.003). In this model, CBF again showed significant differences between MCI and CN (β = –18.8 mL/100 g/min, p < 0.001). Finally, ATT showed a significant increase in Fazekas 3 compared to Fazekas 0 (β = 0.156 s, p = 0.007).

### 3.4 Regional analyses: Vascular territories and AD-specific regions

Regional analyses revealed several associations between cognitive staging and regional Tex and CBF (Supplementary Table 3). Similar to the global analysis, regional Tex differed between CN and SCD in the anterior circulation ROI (β = –30.1 ms, p < 0.001) and the frontal lobe (β = –28.9 ms, p < 0.001). The distribution of Tex values in the frontal cortex between the different cognition groups is shown in Supplementary Fig. 4 as an example. Tex also differed significantly between CN and MCI in all ROIs, except in the posterior cingulate cortex. CBF differences were observed only between CN and MCI, and this was consistent across all ROIs, even after FDR correction. Only anterior circulation and frontal cortex Tex differed with amyloid status (β = –19.0 ms, p = 0.015, and β = –23.37 ms, p = 0.009, respectively), but these differences did not survive FDR correction. In contrast, cognitive staging differences remained significant after FDR correction. Consistent with the global analysis, no significant regional differences in ATT (data not shown) were observed for amyloid and cognition. These results persisted after adjusting for Fazekas scores, as highlighted in Supplementary Table 3.

## 4 Discussion

This study, using a novel BBB-ASL technique, has three main findings. First, Tex and CBF differences between A + and A- individuals were primarily driven by age and sex, with no differences found for ATT. Second, Tex and CBF differed between CN and MCI across most regions, while Tex also differed between CN and SCD. Third, higher cerebrovascular burden was associated with a decrease in Tex values in the early stages (Fazekas 1 and 2), while higher ATT was found only in the group with confluent changes (Fazekas 3). Together, these findings suggest that BBB water exchange, as measured by ASL as Tex, is sensitive to cognitive decline and cerebrovascular burden, and may be more sensitive than CBF or ATT.

The associations found between age and sex and Tex, CBF, and ATT are in line with those reported in previous studies (Mahroo et al., 2023, Chen et al., 2011, Feron, 2023.12.13.571578 (2023)). Faster GM BBB water exchange (lower Tex) with higher age has been shown in previous smaller multi-TE ASL studies in both mice and humans (Mahroo et al., 2023, Ohene et al., 2020), which might be explained by an increased expression of aquaporin-4 with aging (Verheggen, 2020) or by aging-related pericyte deficiency (Armulik, 2010). Our results also show faster BBB water exchange in males than in females, which aligns with previous DCE studies in humans (Moon et al., 2021) and might be explained by hormonal, genetic, and lifestyle factors (Weber and Clyne, 2021). Also in line with the literature, we found that GM CBF declines with age (Chen et al., 2011, Martin, 1991), ATT increases with age (Feron, 2023.12.13.571578 (2023), Asllani, 2009), and females show higher CBF than males(Tomoto, 2023, Liu et al., 2016). Together, these findings provide confidence in the validity of the novel MRI acquisition technique used in this study. Finally, the strong associations of Tex and CBF with age and sex persisted across all analyses, suggesting that these factors primarily drive the observed changes in BBB permeability. This highlights the importance of accounting for age and sex when interpreting BBB-related changes in the context of AD. Although Tex and CBF were moderately correlated with one another, they showed partially distinct patterns in relation to cognitive staging and cerebrovascular burden, suggesting they may reflect overlapping but non-identical physiological processes. Future exploration of Tex and CBF discrepancies may offer physiological insight into BBB function. Additionally, we observed an interaction between age and sex, where females showed a steeper age-related decline in both Tex and CBF than males. Similar patterns have been reported in ASL perfusion studies and have been linked to midlife hormonal transitions and sex differences in vascular reactivity and vascular risk profiles (Ford, 2022). While we do not have the physiological or endocrine measures to further disentangle these mechanisms, this finding emphasizes that age and sex jointly influence BBB-related water exchange and perfusion, and should be taken into account when interpreting group differences in Tex and CBF.

The apparent relation of amyloid positivity with both Tex and CBF seems to be driven by age and sex. Literature shows mixed results regarding the role of BBB dynamics and amyloid, as a recent study found that BBB permeability was not related to amyloid pathology or APOE genotype (Janelidze, 2017). However, another study suggested that BBB dysfunction contributes to the initiation of amyloid deposition (Griffin et al., 2016); and recent work has also shown that BBB water-exchange can be used to monitor neurovascular responses during anti-amyloid therapy, where changes in kw were observed before ARIA-H onset, suggesting a potential role for Tex in treatment and safety monitoring (Uchida, 2025). Additionally, while some studies have linked hypoperfusion patterns to later stages of AD (Ahmadi, 2023); other studies have shown that CBF increases regionally during early amyloid accumulation (Padrela, 2023); followed by a decline as amyloid plaques progress and cognitive impairment worsens (Meng, 2023, Leeuwis, 2017). Our lack of significant findings after adjusting for age and sex may be due to the limited statistical power, given the relatively small number of A + individuals in our study. Future multi-modal comparisons combining BBB-ASL with MRI markers of small-vessel disease (e.g., WMH burden on FLAIR, microstructural damage on DTI) and with amyloid biomarkers (CSF Aβ42/40 or amyloid-PET) will be important to study whether Tex aligns more strongly with vascular or amyloid-related processes. If future studies confirm that Tex is indeed mostly independent of amyloid pathology, it could still (i) enrich trials targeting vascular contributions to cognitive decline, (ii) serve as an early-response marker for vascular risk modification (e.g., blood pressure, glycemic control, or lifestyle interventions), and (iii) identify cerebrovascular involvement in preclinical stages (e.g., SCD), where we observe Tex differences ahead of CBF and ATT***.***

In contrast, ATT did not show any association with amyloid status. To our knowledge, only a single study has evaluated this association and found no difference in ATT between CN and AD groups (Yoshiura, 2009). To what extent Tex, CBF, and ATT are not related to amyloid accumulation or whether their effects are more subtle than our sample size allowed to detect cannot be differentiated with our data. Although our unadjusted associations between amyloid and Tex and CBF are promising, future research with larger cohorts is needed.

Regarding cognition, while both Tex and CBF showed effects between CN and MCI, only Tex showed significant differences already at the SCD stage compared to CN. This finding suggests that BBB water exchange may capture early microvascular alterations associated with cognitive decline, preceding changes in perfusion or macrovascular parameters. This is particularly relevant given that SCD is increasingly recognized as a potential preclinical stage of AD – even suitable for primary intervention (Fladby, 2017) –, where subtle pathophysiological changes, including altered perfusion patterns and increased amyloid burden, have already been reported (Wang, 2020, Thomas, 2021). Also in line with our Tex findings on amyloid, a recent study found that BBB permeability was associated with dementia, but not with amyloid pathology or APOE genotype (Janelidze, 2017). Moreover, our findings are in line with the results of previous contrast agent-based imaging techniques, which reported altered BBB permeability in MCI participants (Sweeney et al., 2018, van de Haar, 2016), despite the molecular size difference between the tracers − gadolinium and blood water. Another BBB technique, Water Extraction with Phase Contrast Arterial Spin Tagging (WEPCAST), also provides a non-invasive BBB proxy measurement, albeit only at the whole-brain level (Lin, 2018). Similar to our findings, a previous WEPCAST study also showed higher BBB water permeability in MCI compared to CN participants (Lin, 2021). However, no study has so far demonstrated BBB water exchange differences between CN and SCD cognition stages.

Additionally, we found a regional decrease in AD-specific regions of Tex between SCD and MCI stages, while CBF only decreases in MCI staging. Although these regional BBB water exchange alterations in such regions have not been reported before, the CBF results were expected based on recent studies that found lower CBF in the hippocampus, precuneus, and orbital frontal cortex using ASL (Dai, 2009, Taghvaei et al.,). Our CBF differences between CN and MCI stages align with previous findings (Leeuwis, 2017, Zhang, 2021). Nevertheless, hyperperfusion has been reported previously in SCD subjects (Thomas, 2021); as well as in the early accumulation regions of amyloid in cognitively unimpaired subjects (Padrela, 2023) and in the frontotemporal areas in subjects at risk of AD (Dounavi, 2023), which may reflect compensatory mechanisms of CBF. The fact that ATT did not differ between the cognitive stages suggests that our Tex associations with cognition are not mainly driven by vascular effects.

The cerebrovascular associations that we found suggest that BBB water exchange, measured by multi-TE ASL, may serve as an earlier biomarker than CBF or ATT. We observed that altered BBB water exchange was already detectable in patients with relatively low cerebrovascular burden (Fazekas 1–2) compared to those without (Fazekas 0), while ATT differences were only evident with severe cerebrovascular burden (Fazekas 3). This is interesting given that ATT is typically considered the strongest (macro-)vascular ASL parameter, having been more consistently associated with age (Feron, 2023.12.13.571578 (2023)), BMI(Feron, 2023), hypertension (Mutsaerts, 2020), and cognitive function in patients with coronary heart disease (Macintosh, 2015) and with small vessel disease(Zhang, 2022). In contrast, we found no associations between CBF and cerebrovascular burden, which differs from previous findings(Bastos-Leite, 2008, van Dalen, 2016, Kim, 2022, Taghvaei, 2025). Gadolinium-based imaging techniques have demonstrated associations between BBB integrity and cerebrovascular burden, suggesting increased permeability in white matter injury(Hergert, 2024). Additionally, there are associations between the progression of WMH and BBB permeability in the basal ganglia(Wardlaw, 2013). The fact that we find similar results using a non-invasive technique is promising for the field. However, Tex is still in the early stages of validation, and further research is needed to establish its robustness. Importantly, while Tex is often interpreted as a marker of BBB water permeability, it is also influenced by the capillary surface area, blood volume and AQP4-mediated water transport at the astrocytic endfeet(Ohene, 2019). Thus, Tex is physiologically distinct from Ktrans (which reflects the bulk leakage of large solutes) as it is sensitive to the subtle physiological regulation of water transport and should be interpreted as a composite measure of microvascular water transport rather than permeability alone. Finally, whether BBB water exchange contributes to cognitive decline indirectly through WMH burden, or whether both reflect independent parallel processes, cannot be accurately modeled from these data. Longitudinal designs with sufficient or larger cross-sectional studies with sufficient power for mediation modeling are encouraged to assess pathways between vascular risk, BBB function, white matter integrity, and cognition.

This study has several potential limitations. First, the cohort was imbalanced in terms of cognitive impairment status, with a smaller number of MCI compared to SCD and CN participants. Another limitation of the multi-TE acquisition technique is that it only models the intravascular and extravascular compartments based on a single parameter (T2), which may oversimplify the interpretation of the BBB water dynamics. While modeling of more compartments is theoretically possible, this is practically limited by the spatial resolution and inherently low signal-to-noise ratio. Nevertheless, this technique has been validated to some extent in both mice and humans, as it was able to detect the effects of aquaporin-4 knockout in mice(Ohene, 2019) and exhibits similar aging-related changes in mice and humans(Mahroo et al., 2023, Ohene et al., 2020, Padrela, 2025). Diffusion-based BBB-ASL techniques have also shown the opposite effect – slower BBB water exchange – with increasing age(Ford, 2022, Morgan, 2024). These discrepancies could be due to the complex, multi-compartmental structure of the BBB, which is often oversimplified into an intra- and extravascular compartment, whereas different extravascular compartments may have distinct T2- or diffusion-weighting properties. Additionally, the BBB Tex model relies on several assumptions, including fixed literature values for blood and tissue T2. Blood T2 varies with oxygenation and may therefore differ across individuals. We cannot validate with our data if this substantially affected our results, but a prior sensitivity analysis using age-adjusted blood T2 values demonstrated minimal impact on the estimated Tex and its association with age(Padrela, 2025). Nevertheless, Tex should be interpreted as a composite measure of BBB water exchange and other physiological contributors, including the oxygen content of the blood***.*** Another limitation lies in the use of slightly different imaging protocols between the two cohorts for the non-BBB measures — for example, amyloid PET versus CSF, and 2D versus 3D FLAIR — which we mitigated by focusing on staging differences rather than examining these measures as continuous variables. To keep our results comparable with previous articles, we used the pre-existing cohort-specific amyloid positivity definitions: 18F-flutemetamol amyloid-PET principal component analysis for LCBC and both 18F-flutemetamol amyloid-PET and CSF Aβ42/40 for the DDI cohort. Even though CSF Aβ42/40 and [18F]Flutemetamol amyloid-PET show high concordance (90–95 %) at the group level in detecting amyloid positivity(Janelidze, 2017, Palmqvist, 2014), they may represent different points in the temporal cascade of amyloid accumulation, with CSF abnormalities generally occurring earlier than PET signal changes. This could partly explain why our Tex differences between amyloid-status groups disappeared after age and sex adjustments. Moreover, although Fazekas ratings provide a well-validated and widely used ordinal index of white matter lesion burden, they do not capture regional lesion distribution or more subtle microvascular changes that may not yet be visible as hyperintensities. Thus, while inter-rater agreement is high (Haughey, 2025); individuals with the same score may differ in the degree of microstructural damage or vascular remodeling that could influence BBB water exchange. Therefore, the relationship between BBB permeability measures and WMH should be interpreted at the group level, and future work with quantitative WMH volume or diffusion-based microstructural metrics may improve sensitivity to vascular contributions.

In conclusion, our findings suggest that the observed BBB water exchange effects are not directly related to AD-specific pathology but reflect more general aging-related neurodegenerative processes, including cerebrovascular damage. BBB alterations have likewise been implicated in other conditions with microvascular involvement, such as genetic small-vessel disease (e.g., CADASIL)(Uchida, 2020) and developmentally regulated vascular maturation(Uchida, 2023); where BBB dysfunction has been linked to altered iron handling and microvascular integrity. Investigating BBB water exchange across a broader spectrum of diseases and comparing multi-modal BBB imaging approaches to assess BBB dynamics could elucidate its potential as a universal or disease-specific biomarker. Finally, these results underline the importance of assessing early cerebral microvascular pathology in aging and neurodegenerative diseases.

## 5 Disclosures

*B.E. Kirsebom* has served as a consultant for Biogen and advisory boards for Eisai and Eli Lilly.

*F. Barkhof* serves on the Steering Committee and Data Safety Monitoring Board for Biogen, Merck, Eisai, and Prothena. Advisory board member for Combinostics, Scottish Brain Sciences, Alzheimer Europe. Consultant for Roche, Celltrion, Rewind Therapeutics, Merck, Bracco. Research agreements with ADDI, Merck, Biogen, GE Healthcare, Roche. Co-founder and shareholder of Queen Square Analytics LTD.

## 6 Consent Statement

All participants provided written informed consent, and the Regional Committee for Medical and Health research Ethics South-East evaluated (based on the Norwegian Health and Research Act and the Helsinki Declaration of 1964; revised 2013) and approved the study. All further study conduct was in line with these guidelines.

## CRediT authorship contribution statement

**Beatriz E. Padrela:** Writing – review & editing, Writing – original draft, Project administration, Methodology, Formal analysis, Data curation, Conceptualization. **Sandra Tecelão:** Project administration, Formal analysis, Data curation, Conceptualization. **Bjørn-Eivind Kirsebom:** Writing – review & editing, Methodology, Conceptualization. **Oliver Geier:** Software, Methodology, Data curation. **Mario Tranfa:** Data curation. **Federico Masserini:** Data curation. **Markus H. Sneve:** Writing – review & editing, Project administration, Methodology, Funding acquisition, Conceptualization. **Maksim Slivka:** Writing – review & editing, Data curation. **Emilie Sogn Falch:** Writing – review & editing, Data curation. **Lene Pålhaugen:** Writing – review & editing. **Amnah Mahroo:** Writing – review & editing, Methodology. **Klaus Eickel:** Writing – review & editing. **David L. Thomas:** Writing – review & editing, Conceptualization. **Matthias Günther:** Writing – review & editing, Methodology. **Per Selnes:** Resources, Project administration. **Atle Bjørnerud:** Project administration. **Kristine B. Walhovd:** Writing – review & editing, Project administration, Funding acquisition, Conceptualization. **Anders M. Fjell:** Writing – review & editing, Project administration, Methodology, Funding acquisition, Conceptualization. **Frederik Barkhof:** Writing – review & editing, Validation, Supervision. **Jan Petr:** Writing – review & editing, Writing – original draft, Validation, Supervision, Methodology, Conceptualization. **Tormod Fladby:** Writing – review & editing, Supervision, Project administration, Funding acquisition, Conceptualization. **Henk J.M.M. Mutsaerts:** Writing – review & editing, Writing – original draft, Validation, Supervision, Project administration, Methodology, Conceptualization.

## Declaration of Competing Interest

The authors declare that they have no known competing financial interests or personal relationships that could have appeared to influence the work reported in this paper.

## Data Availability

Data will be made available on request.
