# Supplementary material for: Blood-brain barrier water exchange in relation to amyloid, cognition and cerebrovascular burden
Source: Neuroimage Clin. 2025 Dec 11;49:103926. doi: 10.1016/j.nicl.2025.103926 (PMC12765162; doi:10.1016/j.nicl.2025.103926)
Supplement: Supplementary Data 1 [file mmc1.docx]

Supplementary materials
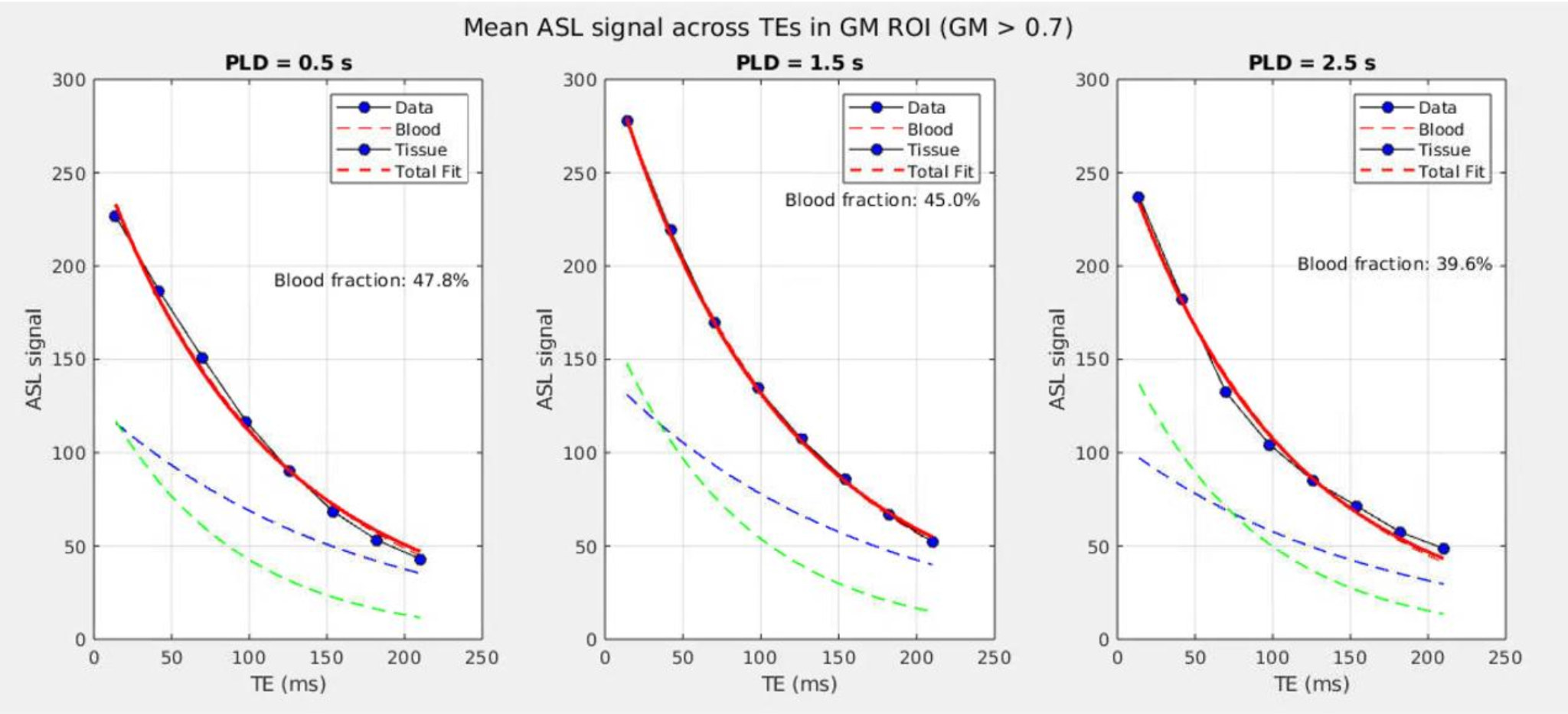


**Supplementary Figure 1:** Representative two-compartment exponential fit to multi-echo ASL signal decay in gray matter at three post-labeling delays (PLDs). Measured ASL signal (black dots) is shown as a function of echo time (TE), with the total fitted signal (red line) overlaid. The model assumes fixed transverse relaxation times for blood (T2 = 165 ms) and tissue (T2 = 85 ms), and estimates the relative contribution of each compartment. The vascular (blood) and tissue signal components are shown as dashed blue and green lines, respectively. The blood fraction decreased with increasing PLD, consistent with progressive water exchange from intravascular to extravascular compartments.

**
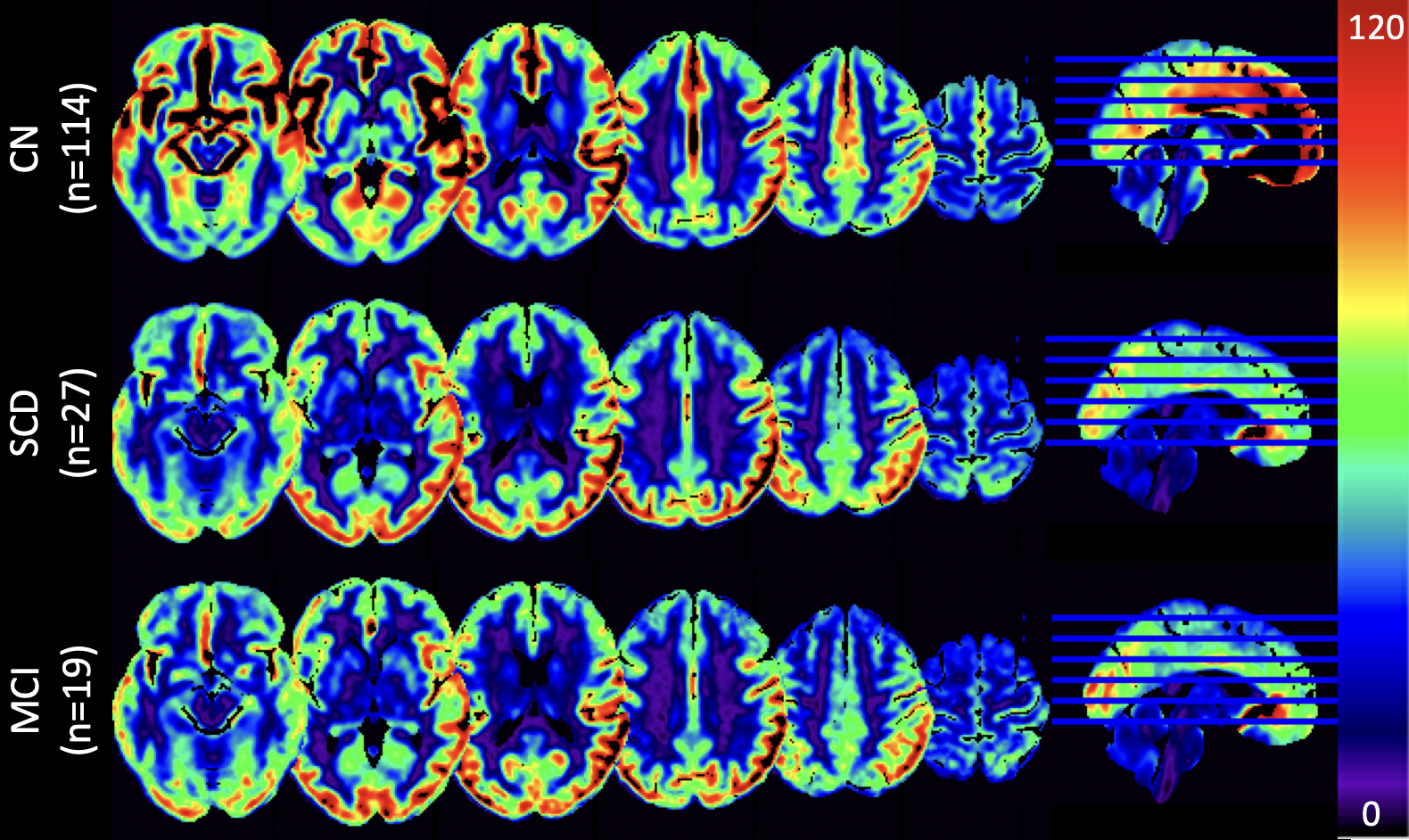
**

**Supplementary Figure 2:** Group-average whole-brain CBF maps for cognitively normal group (n=114, top row); subjective cognitive decline, CSD, n=27, middle row) and mild cognitive impaired group (MCI, n=19, bottom row). Figure done with MRIcron, color scale ‘NIH’, slices [40:10:90].


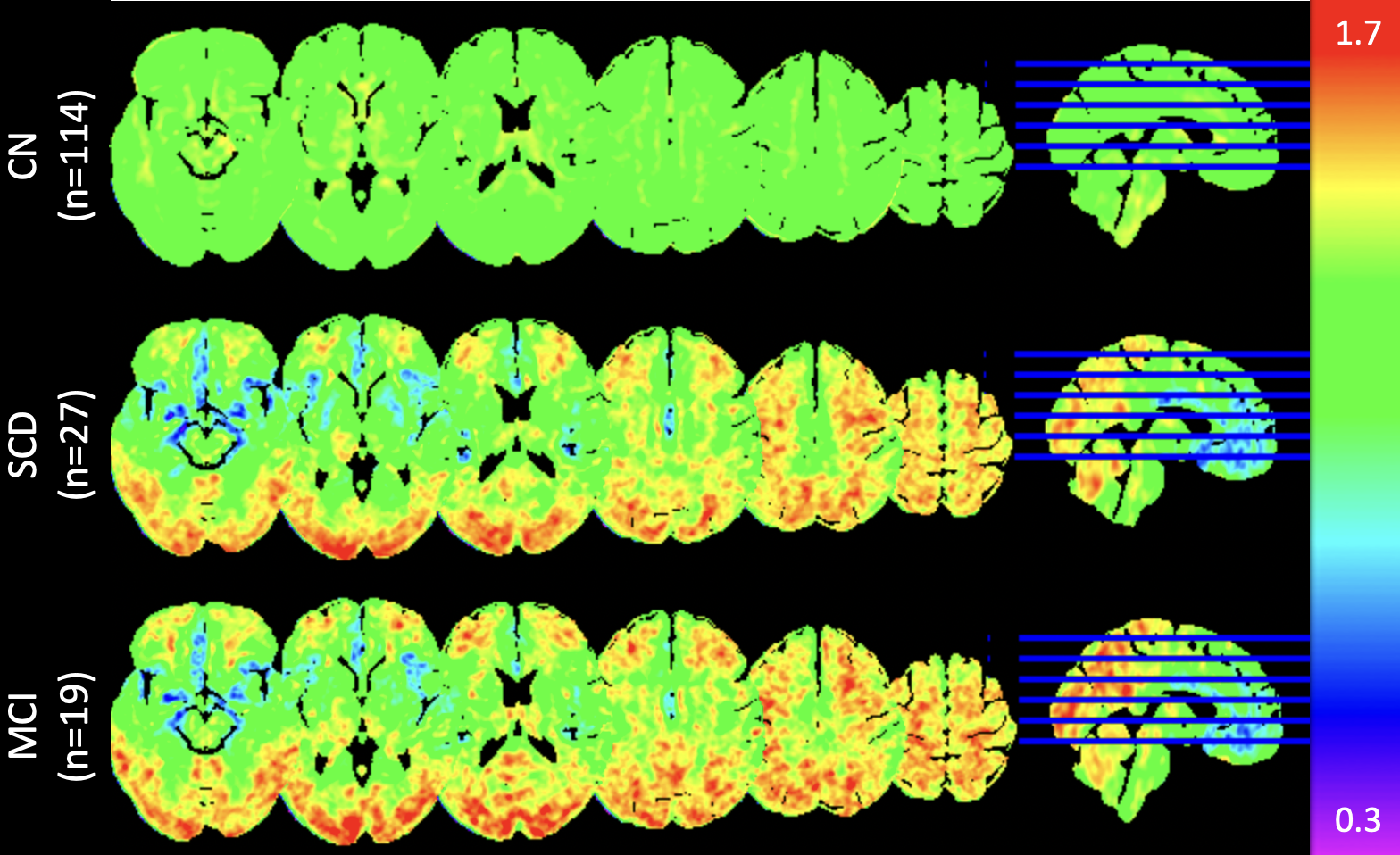


**Supplementary Figure 3:** Group-average whole-brain arterial transit time (ATT) maps for the cognitively normal (CN) (n=114, top row); subjective cognitive decline (SCD) n=27, middle row) and mild cognitive impaired (MCI) groups (n=19, bottom row).


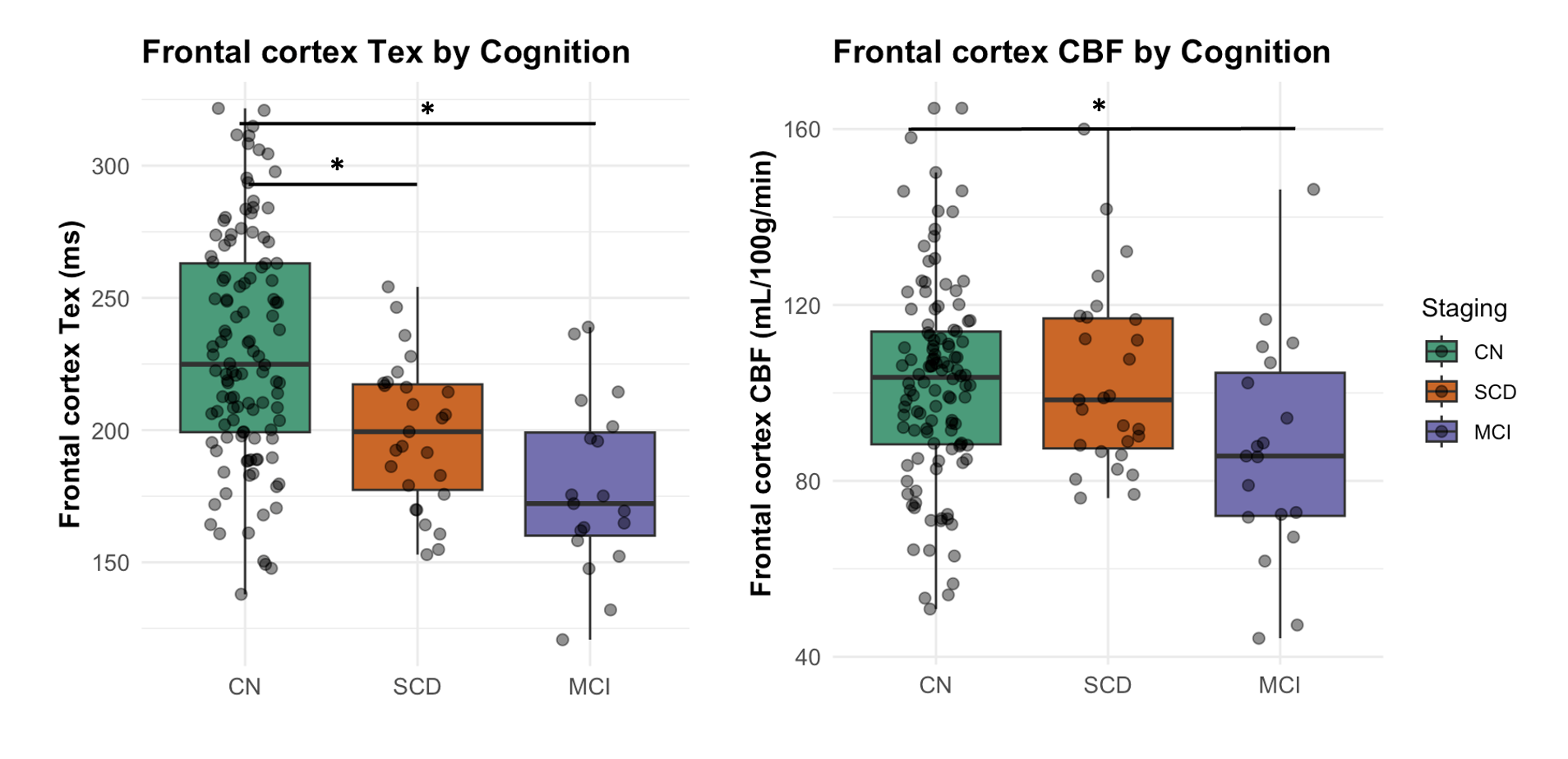


**Supplementary Figure 4:** Differences in cognition staging in Frontal time of exchange (Tex) (left pane) and cerebral blood flow (CBF) (right pane). ANOVA tests with * p<0.05.

**Supplementary Table 1:**  Study sample GM ASL-derived metrics between cognitive staging and amyloid positivity

|  | **A- (n=101)** | **A+ (n=12)** | **CN (n=114)** | **SCD (n=27)** | **MCI (n=19)** |
| --- | --- | --- | --- | --- | --- |
| **Total GM Tex (ms)** |  |  |  |  |  |
| Mean (SD) | 218 (42.1) | 202 (39.6) | 221 (41.5) | 200 (38.9) | 189 (40.3) |
| **Total GM CBF (mL/100g/min)** |  |  |  |  |  |
| Mean (SD) | 87.3 (19.9) | 79.5 (18.9) | 88.8 (19.7) | 81.0 (17.3) | 70.3 (19.1) |
| **Total GM ATT (s)** |  |  |  |  |  |
| Mean (SD) | 1.33 (0.12) | 1.36 (0.09) | 1.34 (0.11) | 1.31 (0.12) | 1.36 (0.12) |

ATT: Arterial transit time; CBF: cerebral blood flow; CN: cognitively normal; GM: gray matter; MCI: Mild Cognitive Impairment; SD: standard deviation; Tex: time of exchange.

**Supplementary Table 2:** Linear models assessing the associations between GM Tex (left), GM CBF (middle), and GM ATT (right) across Amyloid status (top rows), Cognitive staging (middle rows) and Fazekas scorings (lower rows), assessed with and without corrections for age and sex. Statistics for amyloid status are reported relative to amyloid negative (A-), statistics for cognitive staging are relative to cognitively normal (CN), and statistics for Fazekas scores are relative to Fazekas = 0.


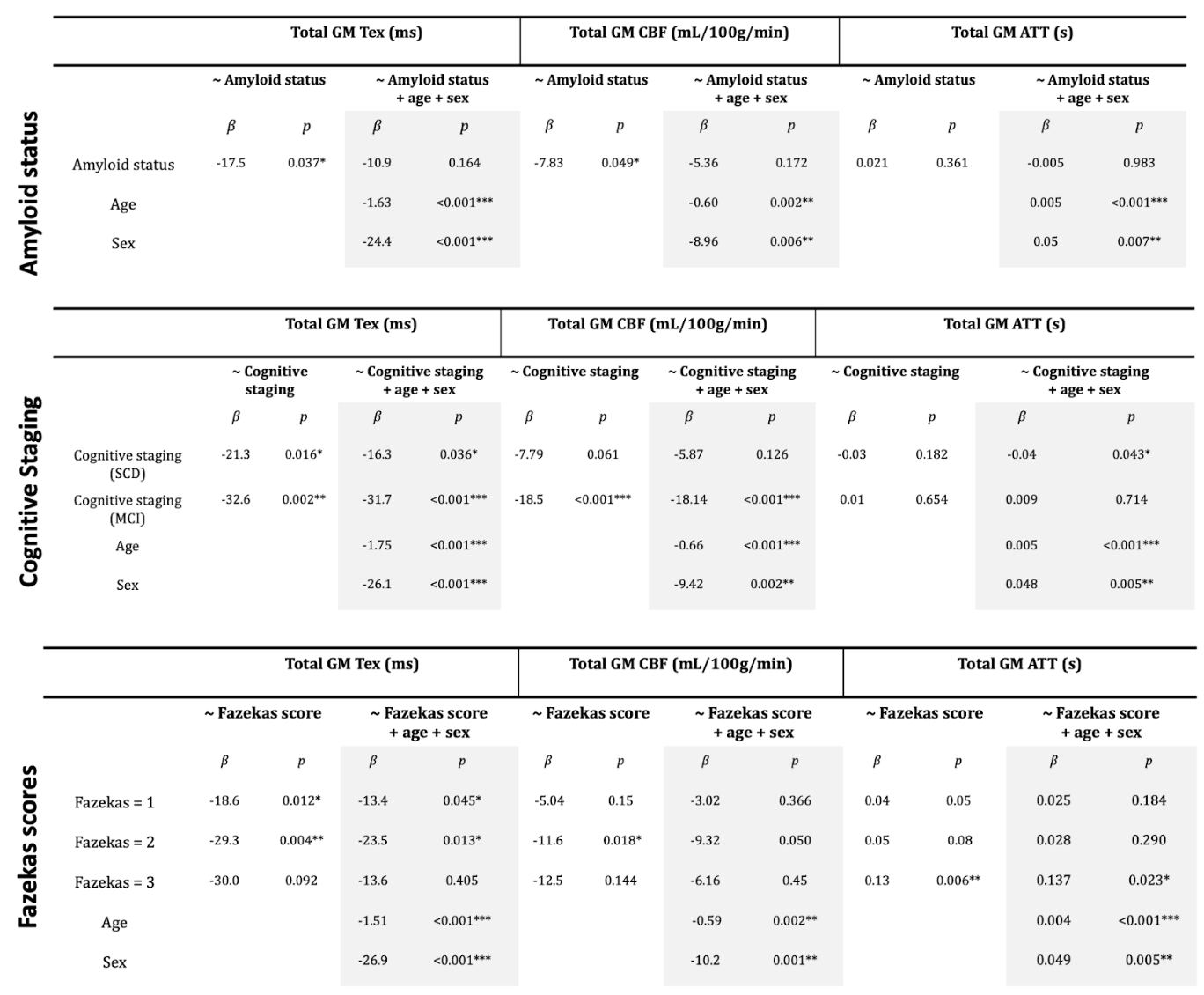


**Supplementary Table 3 -** Results from linear models of regional CBF and Tex correlated with amyloid status and Staging, corrected for age and sex. For amyloid status, the reference group is A- and for Staging, the reference group is CN.

| **Metric** | **Amyloid status** | | **Staging SCD** | | **Staging MCI** | |
| --- | --- | --- | --- | --- | --- | --- |
|  | β | *p* | β | *p* | β | *p* |
| **Vascular Territories Tex** |  |  |  |  |  |  |
| Anterior circulation Tex | –19.0 | 0.015*✝ | –30.1 | < 0.001***✝ | –44.7 | < 0.001***✝ |
| Posterior circulation Tex | –10.0 | 0.292 | –19.8 | 0.036* | –30.2 | 0.006 ** ✝ |
| Ratio Anterior/Posterior Tex | –33.5 | 0.161 | –46.5 | 0.051 | –67.2 | 0.015* ✝ |
| **AD-specific regions Tex** |  |  |  |  |  |  |
| Frontal Tex | –23.37 | 0.009*✝ | –28.9 | < 0.001***✝ | –51.6 | < 0.001*** ✝ |
| Precuneus Tex | –8.79 | 0.398 | –4.78 | 0.649 | –24.7 | 0.043* |
| Posterior cingulate Tex | –14.0 | 0.269 | –15.9 | 0.215 | –26.3 | 0.077 |
| Anterior cingulate Tex | –14.6 | 0.083 | –4.65 | 0.574 | –34.2 | < 0.001*** ✝ |
| **Vascular Territories CBF** |  |  |  |  |  |  |
| Anterior circulation CBF | –6.14 | 0.158 | –2.37 | 0.581 | –19.0 | < 0.001*** |
| Posterior circulation CBF | –4.09 | 0.336 | 1.66 | 0.698 | –10.4 | 0.036* |
| Ratio Anterior/Posterior CBF | –0.02 | 0.497 | –0.05 | 0.067 | –0.09 | 0.008** ✝ |
| **AD-specific regions CBF** |  |  |  |  |  |  |
| Frontal CBF | –4.58 | 0.338 | 2.22 | 0.638 | –15.0 | 0.006**✝ |
| Precuneus CBF | –4.61 | 0.310 | 4.59 | 0.313 | –12.2 | 0.022* |
| Posterior cingulate CBF | –9.47 | 0.075 | 2.54 | 0.627 | –22.11 | 0.001*** |
| Anterior cingulate CBF | –1.51 | 0.778 | 0.613 | 0.909 | –15.9 | 0.011* |
|  |  |  |  |  |  |  |

AD: Alzheimer’s Disease; CBF: cerebral blood flow; GM: gray matter; SD: standard deviation; Tex: time of exchange. * p < 0.05, ** p < 0.01, *** p < 0.001. ✝ Remained significant after correcting for Fazekas scores. CBF is shown in mL/100g/min. Tex is shown in ms.
